# Emerging insights into the type I secretion system: a key player in *Salmonella* virulence and host-pathogen interactions

**DOI:** 10.1128/mbio.01655-25

**Published:** 2025-09-22

**Authors:** Siqi Li, Hongtao Liu, Jiazhang Qiu

**Affiliations:** 1 State Key Laboratory for Diagnosis and Treatment of Severe Zoonotic Infectious Diseases, Key Laboratory for Zoonosis Research of the Ministry of Education, College of Veterinary Medicine, Jilin University 623721, Changchun, China; The Ohio State University, Columbus, Ohio, USA

**Keywords:** *Salmonella*, type I secretion system (T1SS), SiiE, pathogenesis

## Abstract

*Salmonella* spp. are major zoonotic bacterial pathogens that can cause a range of diseases in both humans and animals, including gastroenteritis, septicemia, and typhoid fever. During intestinal colonization, *Salmonella* relies on the coordinated action of the SPI-4 encoded type I secretion system (T1SS) and SPI-1 encoded type III secretion system (T3SS-1) to breach epithelial barriers. Although the T3SS-1 and its effectors have been widely studied, the T1SS and its associated effectors remain poorly characterized. The T1SS-secreted substrate SiiE binds to host cell surface mucins via its bacterial Ig-like (BIg) domain, facilitating the proper positioning of the T3SS-1 and subsequently triggering bacterial internalization. Given the critical role of the T1SS and SiiE in *Salmonella* virulence, they may serve as promising targets for anti-virulence drug development. In this review, we provide an overview of the current knowledge on the T1SS, including its regulatory mechanisms, channel formation, and the functional properties of SiiE.

## INTRODUCTION

The increasing prevalence of antimicrobial-resistant strains and emerging pathogens has significantly exacerbated the global burden of foodborne illnesses, which can progress to severe clinical manifestations including gastroenteritis and systemic sepsis with potentially fatal outcomes (1, 2). Among foodborne pathogens, *Salmonella* spp. represent one of the most clinically significant bacterial threats, primarily transmitted through consumption of contaminated animal- and plant-derived food products as well as water sources (3). *Salmonella* taxonomy currently recognizes over 2,600 serotypes, broadly classified into typhoidal (TS) and nontyphoidal (NTS) strains. While TS serovars are human-adapted pathogens responsible for enteric fever (4), NTS strains demonstrate a remarkably broad host range encompassing humans, poultry, livestock, and wildlife (5). Although most NTS infections present as self-limiting gastroenteritis (6), invasive NTS (iNTS) infections can develop into life-threatening systemic conditions including bacteremia and meningitis, particularly in immunocompromised individuals such as malnourished children, patients with malaria or sickle-cell anemia, and HIV-positive adults (6, 7). Current epidemiological estimates indicate approximately 535,000 annual NTS infections worldwide, resulting in more than 77,500 deaths (7), highlighting the urgent need for improved understanding of *Salmonella* pathogenesis to develop effective control measures.

Recent advances in molecular microbiology have significantly enhanced our understanding of *Salmonella* virulence mechanisms. The remarkable capacity of the pathogen for host cell invasion and intracellular survival is mediated by an extensive repertoire of virulence factors, predominantly encoded within *Salmonella* pathogenicity islands (SPIs). To date, researchers have identified 23 SPIs (8–10), among which SPI-1 and SPI-2 encode the type III secretion systems (T3SS-1 and T3SS-2) that are essential for virulence. T3SS-1 functions as a sophisticated molecular syringe that sequentially delivers effector proteins into host cells. Among these proteins, SipA and SipC manipulate actin dynamics to induce characteristic filopodial-like membrane protrusions at invasion sites (11), while SopE activates Rho GTPases and SopB recruits sorting nexin SNX18 to orchestrate membrane ruffling and facilitate bacterial internalization (12, 13). Following successful invasion, *Salmonella* establishes residence within a specialized membrane-bound compartment termed the *Salmonella*-containing vacuole (SCV), where T3SS-2 effectors (including SifA, SseF, SseJ, and SopD2) mediate SCV remodeling and formation of intricate tubular networks called *Salmonella*-induced filaments (Sifs) that support intracellular replication and survival (14).

While T3SS-1-mediated effector translocation suffices for invasion of nonpolarized epithelial cells, penetration of polarized epithelial cells requires additional virulence factors. Mucins secreted by goblet cells and other intestinal epithelial cells establish a mucus layer that is able to separate the epithelial surface from the lumen (15). In addition, the distinct compositions of apical and basolateral sides within polarized cells can close cell contacts owing to the formation of tight junctions (16). These features allow the intestine to control nutrient absorption, while forming a physical barrier that prevents invasion by pathogenic bacteria. To overcome these barriers, *Salmonella* employs the SPI-4-encoded type I secretion system (T1SS) to secrete SiiE, a giant non-fimbrial adhesin that plays a pivotal role in intestinal colonization (17, 18). The 595 kDa protein extends from the bacterial surface to engage specific glycoprotein receptors on polarized intestinal epithelial cells (19, 20), establishing the critical bacterium-host contact required for subsequent T3SS-1 effector translocation and successful invasion (21).

Growing evidence underscores the crucial role of the T1SS and SiiE in *Salmonella* pathogenesis, particularly during the initial stages of intestinal colonization. This review systematically synthesizes current knowledge regarding *Salmonella* invasion strategies for polarized epithelial cells, with particular emphasis on the following: (i) the molecular architecture and regulatory mechanisms of T1SS; (ii) structural and functional characteristics of its secreted effector SiiE; (iii) mechanistic insights into SiiE-mediated adhesion and invasion processes. Furthermore, we provide a comprehensive analysis of SiiE binding properties and discuss potential therapeutic implications of targeting this virulence factor.

## TYPE I SECRETION SYSTEM (T1SS) IN BACTERIA

Gram-negative bacteria have evolved a remarkable array of sophisticated nanomachines to transport various virulence factors across their cell envelope. To date, ten distinct secretion systems (T1SS–T6SS and T8SS–T11SS) have been characterized for their ability to secrete diverse protein substrates (22–24). Notably, while the T7SS has been identified, it is exclusively found in gram-positive bacteria and pathogenic mycobacteria (25). Among these secretion systems, the T1SS exhibits the simplest structural organization, comprising three essential components: (i) an ATP-binding cassette (ABC) transporter that forms the inner membrane channel; (ii) an outer membrane protein (OMP) that constitutes the outer membrane channel; (iii) a membrane fusion protein (MFP) that bridges the ABC transporter and OMP components (26). T1SS substrates display considerable size variation (ranging from 20 kDa to 1.5 MDa) and functional diversity, including heme-binding proteins, adhesins, pore-forming toxins, and lipases (27–29). One of the well-studied T1SS substrates is the pore-forming RTX (Repeats in Toxin) toxin hemolysin A (HlyA). HlyA is specifically transported by a T1SS composed of the ABC transporter HlyB, the OMP TolC, and the MFP HlyD (30). The 1,024-amino acid *Escherichia coli* alpha-hemolysin contains characteristic glycine-rich repeats (GG repeats) that define the RTX domain (31). These repeats create multiple calcium-binding sites that facilitate HlyA folding in the extracellular environment, resulting in its biologically active conformation (32). Although all RTX proteins contain C-terminal secretion signals, these sequences show minimal conservation among different RTX family members (26). In HlyA, the secretion signal is localized to the C-terminal 48–60 amino acid residues (33).

Since its initial discovery in *E. coli*, the T1SS has been detected in more than half (54%) of all sequenced gram-negative bacterial genomes (34), indicating its fundamental importance in bacterial physiology and pathogenesis.

## T1SS ENCODED BY SPI-4 IN *SALMONELLA*

SPI-4 was identified as a 24 kb *Salmonella*-specific genomic region that is essential for bacterial survival within macrophages, attributed to the presence of the *ims98* locus (17). Notably, SPI-4 genes, along with SPI-1 genes, are downregulated during macrophage infection. SPI-4 mutants show attenuated virulence during oral infection in mice while still causing systemic salmonellosis following intraperitoneal inoculation (35–37). These findings indicate that SPI-4 is crucial for *Salmonella* gastrointestinal infection. Gerlach et al. demonstrated the critical role of SPI-4 in epithelial cell adhesion using MDCK cells, which reliably forms polarized epithelia (18).

**Fig 1:**
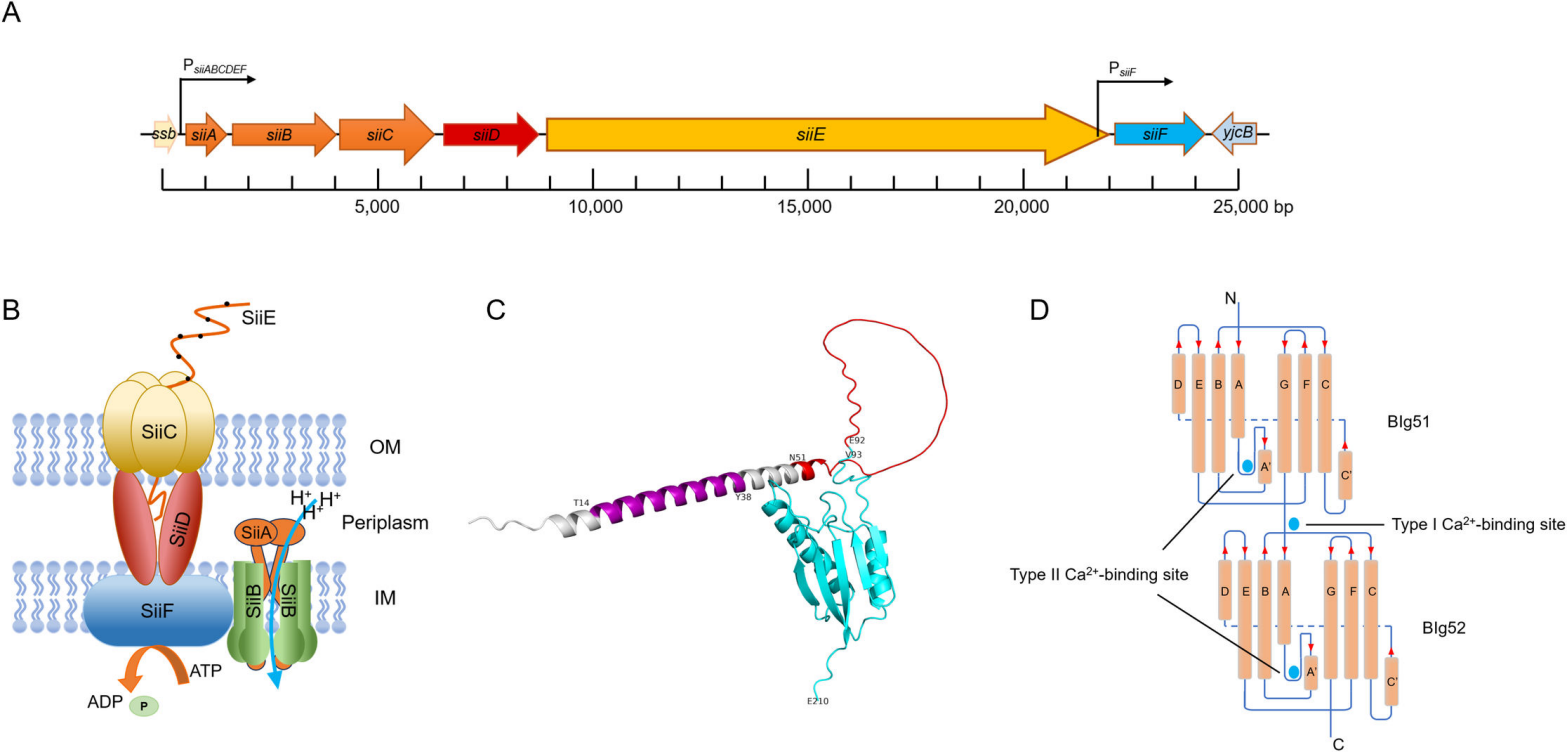
(**A**) Schematic representation of the SPI-4 locus in *S.* Typhimurium. SPI-4 is a 24 kb pathogenicity island containing six OFRs (*siiA* to *siiF*)*,* flanked by the gene *ssb* (upstream) and the gene *yjcB* (downstream). In the schematic, ORFs are depicted as colored arrows oriented in the direction of transcription, with black curved arrows indicating promoter regions near the transcription start sites. Each space represents 1,000 bp, with a total of 25 spaces (25,000 bp). (**B**) Schematic diagram of the T1SS in *S.* Typhimurium. The diagram illustrates the core subassemblies of the T1SS and their spatial arrangement relative to the outer membrane (OM) and inner membrane (IM). The SiiA and SiiB proteins assemble into the SiiAB transmembrane complex, forming a proton-conducting pore channel. The ABC transporter SiiF, localized on the cytoplasmic side of the IM, provides energy for substrate translocation through ATP hydrolysis. SiiD functions as a bridging adapter protein, physically coupling the SiiF transporter to the OM β-barrel protein SiiC, thereby establishing a continuous secretion conduit across the bacterial envelope. (**C**) Domain architecture of the SiiA protein. SiiA consists of three distinct structural domains: transmembrane domain (purple; residues 14–38), a flexible, intrinsically disordered periplasmic linker (red; residues 51–92), and a structured globular periplasmic domain (cerulean; residues 93–210). (**D**) Schematic representation of the Ca²^+^-binding site in the BIg domains of SiiE. The topology plot of BIg 51 and 52 in SiiE reveals two distinct Ca^2+^-binding sites on BIg domains. The Ca²^+^-binding sites are marked by cerulean solid circles, either within the β-sheet framework or at the interface between two BIg domains. The orientation of β-strands is indicated by red solid triangles (N- to C-terminal polarity).

SPI-4 contains six open reading frames (ORFs) designated *siiA-F* (Fig. 1A), among which SiiA, SiiB, SiiC, SiiD, and SiiF constitute cognate T1SS responsible for the secretion of SiiE, a giant non-fimbrial adhesin (Fig. 1B). Furthermore, *Salmonella* T1SS secretes BapA, a large cell-surface protein essential for biofilm formation, thereby underscoring the remarkable substrate diversity of this secretion machinery (38).

### SiiC, SiiD, and SiiF: the type I secretion apparatus

SiiC, SiiD, and SiiF constitute the canonical building blocks of the T1SS: the ABC transporter SiiF, the membrane fusion protein SiiD, and the outer membrane protein SiiC (18). Together, they form a continuous transmembrane-spanning tunnel that facilitates one-step transport of substrates to the extracellular environment, bypassing periplasmic intermediates. Importantly, the secretion function T1SS substrate is abolished when any of *siiC*, *siiD*, or *siiF* is mutated (37).

Generally, SiiF-like ABC transporters contain two transmembrane domains (TMDs) and two nucleotide-binding domains (NBDs, also termed ATPases or ABCs) (39). ABC proteins characteristically dimerize upon ATP binding (40). Bacterial two-hybrid assays demonstrated that SiiF forms stable complexes with both SiiB and itself, a process dependent on the Walker A motif within its NBD (41). Moreover, in contrast to the ABC transporter PrtD of *Dickeya dadantii*, which lacks an N-terminal auxiliary domain, SiiF contains an additional N-terminal C39 peptidase-like domain (CLD) (42). The N-terminal domain of SiiF shows >98% homology with that of *E. coli* HlyB (43). In HlyB, the CLD (lacking peptidase activity) regulates HlyB activity and is essential for HlyA secretion (44, 45). To date, the function of the N-terminal domain of SiiF remains uncharacterized.

SiiD, a membrane fusion protein (MFP) and periplasmic adapter protein (PAP), connects SiiF and SiiC (43). Structural predictions indicate SiiD shares homology with *E. coli* MacA, AcrA, and HlyD; *Streptococcus pneumoniae* Spr0693; and *Aquifex aeolicus* EmrA (43). Like AcrA and MacA (PAPs in *E. coli* drug efflux pumps), SiiD likely contains three subdomains: a β-barrel domain, a lipoyl domain, and an α-helical domain (46). While SiiD (residues 43–300) has been crystallized, its structural analysis remains incomplete (43). Notably, SiiD was recently shown to inhibit NLRP3 inflammasome activation during *Salmonella* infection, suggesting that T1SS not only drives gut-specific pathogenesis but also potentially mediates immune evasion at systemic sites through its translocation (47).

### SiiA and SiiB: the auxiliary proteins involved in proton translocation across the inner membrane (IM)

Although the secretion of SiiE protein into the extracellular supernatant through the T1SS remained unaffected upon deletion of *siiA* and *siiB*, the ability to invade polarized cells and virulence in mice were significantly diminished (37, 41). This demonstrates that SiiA and SiiB are likely regulatory proteins encoded by SPI-4. SiiA and SiiB belong to the Mot/Exb/Tol family of membrane proteins. They assemble into the SiiAB complex on the IM, where it functions as a proton channel to mediate proton translocation (41, 48). SiiA consists of three distinct regions: a transmembrane domain (aa 14–38), an intrinsically disordered periplasmic linker region (aa 51–92), and a folded globular periplasmic domain (SiiA-PD, aa 93–210) (Fig. 1C) (48). The monomer crystal structure of SiiA-PD reveals a central β-sheet formed by five β-strands and shows structural homology with numerous peptidoglycan (PG)-binding proteins. SiiA binds PG in a pH-dependent manner that requires arginine residues at positions 162 and 167. Notably, mutation of arginine 162 in SiiA markedly reduces *Salmonella* invasion (48). However, the mechanism by which SiiAB utilizes proton motive force to coordinate SiiE retention and release remains unclear (48).

## SiiE-NON-REPEATS IN TOXINS (RTX) Ca^2+^-BINDING ADHESINS

### The Ca^2+^-binding bacterial immunoglobulin (BIg) repeats of SiiE

SiiE, a 595 kDa protein secreted by the T1SS and a member of the non-RTX Ca^2+^-binding proteins, is the largest known protein in *Salmonella* (49, 50). RTX proteins are defined by tandemly repeated nonapeptides enriched in glycine and aspartate, exhibiting the consensus sequence GGxGxDxUx. In this consensus sequence, x denotes any amino acid, whereas U represents a large hydrophobic residue (51). Representative RTX proteins include CyaA from *Bordetella pertussis*, AprA from *Pseudomonas aeruginosa*, and FrpC from *Neisseria meningitidis* (52–54). All RTX proteins share a defining feature: the presence of a non-cleaved C-terminal secretion signal, which is critical for their secretion via the T1SS (32). In contrast, SiiE lacks the RTX repeats sequences and comprises three distinct structural domains: the N-terminal domain, the BIg repeats, and the C-terminal signal sequence (19).

The BIg repeats of SiiE are composed of 53 highly similar repeats of BIg domains, which are distinct from the Ca^2+^-binding RTX repeats (19). The length of SiiE is directly proportional to the number of BIg domains, while a functionally uncharacterized short insertion sequence is observed between BIg52 and BIg53 (19). Deletion of varying numbers of BIg domains does not affect SiiE secretion but reduces its surface retention on the bacterial envelope, thereby impairing invasion (19). Thus, SiiE adhesion to polarized cells depends on the number of its BIg repeats. The C-terminus contains the secretion signal for SiiE (18). The N-terminal domain consists of a coiled-coil (CC) domain formed by eight heptad repeats sandwiched between two β-sheet structures, requiring the integrity of both the CC domain and β-sheet domains for proper SiiE retention and release (19, 43).

The crystal structure of BIg domains 50–52 of SiiE reveals two distinct Ca^2+^-binding sites on the BIg, designated as type I and type II Ca^2+^-binding sites (Fig. 1D). Ca^2+^ binding rigidifies the BIg repeats, reducing molecular flexibility and enhancing resistance to proteolytic cleavage (55, 56). The type I Ca^2+^-binding sites are located at interfaces between two BIg domains, where three aspartate residues from two consecutive BIg domains coordinate Ca^2+^. In contrast, type II Ca^2+^-binding sites are situated within single domains, involving two aspartate side chains. Each BIg domain in SiiE can bind no more than two Ca^2+^ ions (55). Sequence alignment of SiiE revealed 18 positions that are highly conserved across at least 80% of all BIg domains, including six invariant aspartate residues (16D, 24D, 43D, 86D, 97D, and 117D) (55). Among these conserved residues: 16D and 24D serve as signature residues for type II Ca^2+^-binding sites and are present in all domains except domain 50. 43D, 97D, and 117D participate in type I Ca^2+^-binding, while 86D appears unrelated to Ca^2+^ coordination, and its function remains unknown (55). The functional importance of these Ca^2+^-binding sites was demonstrated through mutational analysis. Deletion of Ca^2+^-binding sites in the C-terminal region (BIg47-52Δ10; Δn represents the number of mutated Ca^2+^ binding sites) severely impaired both SiiE secretion and bacterial invasion, with effects comparable to those of an *siiF*-deficient strain (56). In contrast, the secretion levels of the of N-terminal mutation (BIg1-5Δ10) remained significantly higher at 6 hours compared to 3.5 hours post-subculture, indicating that these conserved residues critically regulate SiiE release kinetics (56). Notably, removal of Ca^2+^-binding sites from BIg40 had no detectable effect on the secretion, surface retention, or invasion capabilities of SiiE (56). Further characterization revealed that type I and type II Ca^2+^-binding sites play distinct roles. The number of functional type I sites correlates with secretion efficiency, while type II sites are essential for maintaining proper protein conformation required for adhesion (56).

### The binding properties of SiiE

*Salmonella* utilizes three primary mucosal invasion pathways: M cell translocation, direct epithelial cell invasion, and dendritic cell uptake (57). Surface attachment represents a crucial initial colonization step. SiiE-mediated invasion specifically targets polarized epithelial cells, presumably through receptor recognition. Structural analysis using the Dali server revealed that the BIg50-52 domains of SiiE share homology with adhesion proteins involved in protein-protein and protein-carbohydrate interactions (55). In line with this, Wagner et al. demonstrated the lectin-like binding specificity of SiiE for α 2,3-linked sialic acid through deglycosylation and lectin blockade experiments (58). These findings collectively demonstrate that SiiE interacts with carbohydrate structures in a lectin-like manner (58).

In the gastrointestinal tract, transmembrane mucins (MUC1, MUC3A/B, MUC4, MUC12, and MUC13) form a glycosylated barrier against pathogens (59). While MUC1 typically protects against invasive bacteria like *C. jejuni* and *H. pylori* (60, 61), *Salmonella* exploits it as an SiiE receptor for intestinal epithelial invasion (20). Conversely, MUC13 acts as a decoy receptor that sheds post-*Salmonella* binding to prevent epithelial adhesion and maintain intestinal barrier integrity (62). Additionally, SiiE contains a sulfite oxidase (SO) domain that neutralizes immune cell-derived sulfite, subverting host antimicrobial defenses (63). Further studies are needed to fully characterize the molecular mechanisms and host receptors involved in SiiE-mediated pathogenesis.

## REGULATION OF T1SS

**Fig 2:**
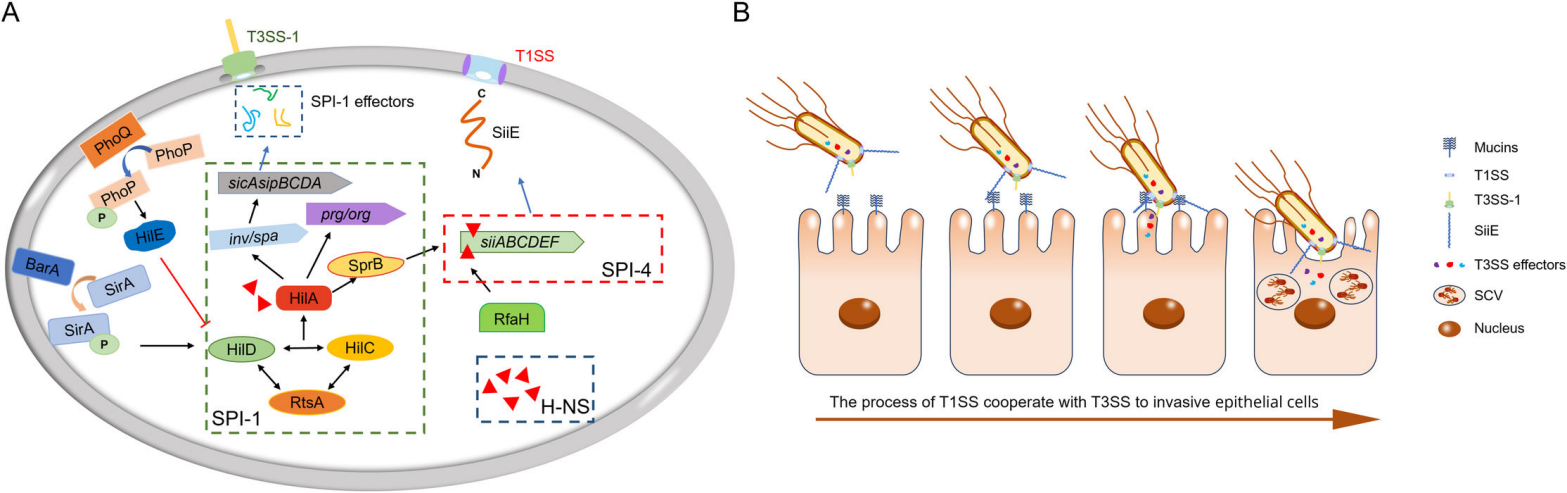
(**A**) Regulation of *S.* Typhimurium SPI-1 and SPI-4. See text for details. *prg/org* and *inv/spa*, which were not explicitly described in the text, denote the promoter regions of the T3SS structural genes, whereas *sicAsipBCDA* represents the promoter of the T3SS effector operon. The black arrows indicate activation, the red line with flat ends represents inhibition, the blue arrows indicate protein translation events, and the elongated arrow denotes the promoter region. Other symbols: red solid triangles (H-NS), thick orange solid line (unfolded SiiE), curved arrow (phosphorylation). (**B**) Invasion process of *S.* Typhimurium trough coordinated action of T1SS and T3SS-1. Schematic sequence: (i) chemotaxis of bacteria toward epithelial cell surfaces; (ii) the BIg domain in SiiE mediates interacts with mucins to facilitate adhesion to epithelial cells; (iii) bacteria employ T3SS-1 to inject effector proteins into the cytoplasm; (iv) cytoskeletal rearrangement induces membrane ruffling, facilitating bacterial internalization and the formation of SCVs.

The activity of the T1SS in *Salmonella* is tightly regulated by an intricate network of environmental signals and transcription factors. Notably, the T1SS and T3SS-1 share a common regulatory circuit, in which the expression of SPI-4 is positively regulated by the transcriptional activators SirA, HilA, HilC, and HilD, while being negatively modulated by HilE, PhoP, and the histone-like nucleoid-structuring protein (H-NS) (Fig. 2A).

Mutations in *hilA* and *sirA* impair SiiE-mediated epithelial cell invasion, with SirA regulating SPI-4 expression in a HilA-dependent manner (64). HilA, an OmpR/ToxR family transcription factor encoded within SPI-1, governs the expression of multiple virulence genes, including *invF*, structural components of the T3SS-1 apparatus, and secreted effector molecules (65). Its activation relies on a positive-feedback loop involving HilD, HilC, and RtsA (66). However, *hilA*, *hilC*, or *hilD* alone cannot activate *siiE* expression in the absence of the SPI-1 locus, suggesting the involvement of an additional HilA-dependent regulator within SPI-1 (67). Studies by Saini and colleagues reveal that SprB, a transcriptional regulator encoded by SPI-1, directly activates the *siiA* promoter. This discovery highlights SprB as the critical molecular connection that orchestrates gene expression across SPI-1 and SPI-4 (68, 69). The PhoP/PhoQ two-component system suppresses SPI-1 function by downregulating *hilA* (70), while HilE acts as a negative regulator by directly interacting with HilD (71). Furthermore, an operon polarity suppressor (ops) sequence within SPI-4 is essential for RNA polymerase (RNAP) pausing and RfaH loading, a critical step in SPI-4 transcriptional regulation (67, 72). Notably, H-NS binds across all SPIs, including SPI-4, but its repressive activity is counteracted by HilA (67). Beyond these core regulators, SPI-4 expression is further influenced by diverse factors, including spermidine, the biofilm-associated gene *yeaM*, the ATP-dependent protease Lon, and the colonization-related gene *yqiC* (73–76). Nevertheless, the precise molecular mechanisms governing these regulatory interactions remain to be fully elucidated.

## CONCLUSIONS AND FUTURE PERSPECTIVES

*Salmonella* pathogenesis involves a multistep process: reaching host cells, adhering to epithelial surfaces, and employing invasion strategies to establish infection. Adhesion is critical not only for colonization but also for providing a stable platform to deliver virulence factors. The giant non-fimbrial adhesin SiiE plays a pivotal role by binding to transmembrane mucins via a lectin-like interaction, enabling *Salmonella* to penetrate the mucin barrier and properly localize the T3SS-1 (20, 58, 62). Upon reaching the inner mucus layer, the BIg domains of SiiE engage with glycosylated tandem repeats, shortening the distance between the bacterium and host cell surface. This binding facilitates T3SS-1-mediated injection of effector proteins, triggering cytoskeletal rearrangements, membrane ruffling, and bacterial internalization (Fig. 2B). The interplay between bacterial secretion systems plays a critical role in facilitating the rapid colonization of host cells, as exemplified by *Salmonella*. Such cooperative mechanisms are not unique to *Salmonella* but are also observed in other pathogens. For example, in *E. coli*, the T3SS delivers the inner membrane receptor Tir, which directly interacts with the T5SS substrate protein intimin to enhance bacterial adherence to host cells (77). Similarly, in the plant pathogen *Dickeya dadantii*, synergistic interactions between the T1SS and T2SS are evident. Specifically, the T1SS-secreted protease PrtA processes the T2SS substrate pectate lyase PelI-2 into its active form, PelI-3, thereby inducing necrosis in tobacco leaves (78). Collectively, these examples underscore the importance of cross-talk between diverse secretion systems in driving bacterial pathogenesis.

### Structural and functional insights of T1SS

Despite its importance, structural characterization of the *Salmonella* SPI-4-encoded Type 1 Secretion System (T1SS) remains elusive, with no available cryo-EM or electron tomography data. However, functional inferences can be drawn from homologous systems, such as the *E. coli* T1SS (29). SiiE, a massive, repetitive adhesin (53 BIg domains), is secreted via the T1SS in a C-terminal-first manner (18, 19, 36). Extracellular Ca^2+^ induces folding of its BIg domains, which harbor two distinct Ca^2+^-binding sites: conserved Type I sites and SiiE-specific Type II sites (55). Intriguingly, SiiE secretion is stalled at the bacterial surface via its N-terminal domain, ensuring prolonged host contact (55).

SiiE-driven colonization exhibits striking host specificity, promoting robust intestinal adherence in cattle but not chickens (50, 79). This discrepancy likely stems from interspecies variations in transmembrane mucins (80, 81). Additionally, the elongated structure of SiiE enables it to protrude beyond long-chain LPS, facilitating host interactions (21). Beyond adhesion, SiiE subverts humoral immunity by selectively depleting IgG-secreting plasma cells, highlighting its multifunctional role in infection (82).

### T1SS as an antimicrobial target

Antimicrobial resistance (AMR) remains a global public health crisis. Currently, antibiotic resistance in *Salmonella* continues to escalate, with surveillance data showing that at least 75% of clinical isolates from Africa exhibit resistance to first-line antibiotics (83). Particularly concerning is the emergence and spread of mobile colistin resistance (*mcr*) genes among *Salmonella* strains, which has significantly exacerbated the problem of multidrug resistance (MDR) (84, 85). This development has severely limited available treatment options for *Salmonella* infections, posing major challenges for clinical management. In recent years, the anti-virulence strategy has gained great interest in the fight against bacterial infections by disarming bacterial virulence factors or pathogenicity. Owing to its the critical roles in *Salmonella* infection and conservativeness among pathogenic serovars of *Salmonella* (86, 87), T1SS may serve as an attractive drug target for anti-virulence therapy. Further discovering of chemicals that inactivate T1SS might provide alternative treatment options for *Salmonella* infection.

### Key unresolved questions of T1SS in *Salmonella*

Recent progress has notably deepened our understanding of the T1SS and its substrate protein SiiE in *Salmonella*. Nevertheless, pivotal questions remain unresolved. Key areas of investigation include the following: (i) the specific roles of individual T1SS components during secretion; (ii) the precise sequential mechanism of T1SS assembly; (iii) the structural characterization of T1SS via advanced techniques such as cryo-EM or electron tomography (ET); (iv) the functional importance of each BIg domain in SiiE; and (v) the regulatory factors governing T1SS activity. Given the critical role T1SS plays in the pathogenesis of *Salmonella*, addressing these unresolved questions is vital for shedding light on its virulence mechanisms. As cutting-edge technologies and analytical methodologies continue to advance, it is plausible that these challenges will be systematically tackled in the foreseeable future, potentially leading to significant breakthroughs in combating *Salmonella*-related diseases.
